# Importance of the diameter of the mechanical suture in rectal surgery in relation to benign anastomotic stenosis. Cross-sectional observational study

**DOI:** 10.1007/s10151-025-03157-9

**Published:** 2025-06-18

**Authors:** N. Llorach-Perucho, L. Cayetano-Paniagua, X. Serra-Aracil

**Affiliations:** 1 Coloproctology Unit, Parc Taulí University Hospital, Institut d’Investigació i Innovació Parc Taulí (I3PT-CERCA), Sabadell, Spain; 2 Coloproctology Unit, Consorci Sanitari de Terrassa, Barcelona, Spain; 3 Department of Surgery, Universitat Autònoma de Barcelona, 08193 Bellaterra, Barcelona Spain

**Keywords:** Anastomotic stenosis, Rectal surgery, Circular stapler diameter, Colorectal anastomosis, Intraoperative dilation

## Abstract

**Background:**

Postoperative benign anastomotic rectal stenosis (BAS) has a significant incidence rate (2–30%). Recently, it has been shown that its incidence decreases with a larger anastomotic diameter (≥ 31 mm). The level of awareness of this data and the interest in creating an intraoperative anastomotic dilation system remain unknown. The aim of the study is to evaluate, using a survey sent to Spanish colorectal surgeons, the knowledge of postoperative strictures in rectal surgery as well as the use of methods to prevent them.

**Methods:**

An observational cross-sectional study was conducted using a survey sent to 101 colorectal surgeons from 49 colorectal surgery units in Spanish hospitals in June 2024.

**Results:**

Eighty-seven responses were obtained (86.1%); 39 (44.8%) were aware of their BAS rate, 41 (47.1%) recognized it as similar to the rate reported by our group (16.3%), and 82 (94.3%) considered this rate too high. Regarding mechanical sutures, none used 25-mm sutures, 43/87 (49.4%) used 28–29-mm sutures, 39/87 (44.8%) used 31-mm sutures, and only 5/87 (5.7%) used 33-mm sutures; 72.4% (63/87) were unaware of the existence of dilation mechanisms, while 15 (17.2%) knew about or used some type of device. In an ideal dilation situation, mechanical dilation (60%) predominated over pneumatic, although the same number of surgeons would choose to use dilators (21/87) as would opt not to use them (22/87). Forty-three of 87 (43.9%) would tend to use larger anastomotic diameters (31 mm).

**Conclusions:**

There is a significant lack of knowledge about the incidence of BAS and its relationship with the anastomotic diameter. Increased awareness of these issues is needed, aiming to use the widest possible mechanical sutures (> 31 mm) and considering the need for dilation devices to reduce the incidence of BAS.

## Introduction

In surgeries of the left colon and rectum, end-to-end anastomoses performed after intestinal resection can present early complications, such as dehiscence and bleeding, as well as late complications, such as benign anastomotic stenosis (BAS).

Despite technological advances and improvements in surgical techniques, complications of left colon and rectal anastomoses remain at 2–30% (with an acceptable risk of 3–6% in the hands of experienced surgeons) [1, 2].

Beyond the impact on the initial postoperative period, anastomotic leakage can have long-term consequences. Its presence increases the risk of permanent stoma, anorectal dysfunction, anastomotic stricture, and local recurrence [3, 4].

BAS has a highly variable incidence (2–30%) due to the limited studies in the literature and the lack of consensus on its definition [1, 2, 5]. One of the most objective classifications of BAS was proposed by Truong [6], who divided it into three grades: grade I, with an anastomotic diameter between 10–20 mm and/or occasional abdominal symptoms; grade II, with an anastomotic diameter between 5–9 mm and/or frequent abdominal symptoms; grade III, with an anastomotic diameter < 5 mm and/or the presence of abdominal symptoms indicating intestinal obstruction.

Seventy percent of colorectal stenoses occur after rectal surgeries, making this type of surgery a risk factor for developing stenosis. Other described risk factors include male sex, obesity, smoking, diabetes, neoadjuvant treatment, type of anastomosis, and its diameter [7, 8]. Concerning the latter point, few studies have demonstrated this relationship. Reif de Paula et al. [9] observed a correlation between the diameter of the mechanical circular stapler and BAS in surgeries of the left colon and rectum.

Our group has recently conducted a study on BAS in low anterior resection (LAR) of the rectum in patients with middle and lower rectal neoplasia (< 10 cm from the anal margin), finding that the BAS rate after LAR is not negligible, occurring in 39 of 239 patients (16.3%). Using a 31–33-mm mechanical circular stapler (MCS) is associated with a lower incidence of BAS, so it is recommended to use larger diameter MCSs [10].

Regarding preventive maneuvers for BAS, to our knowledge, there are no bibliographic references in the literature about the diameter of colorectal anastomoses and the effectiveness, precision, and standardization of mechanical dilation devices available on the market.

Therefore, the aim of this study was to develop a survey to address these issues and assess the feasibility of developing solutions that allow for bigger diameter colorectal anastomoses.

## Methodology

A survey was created and sent via email to 101 Spanish colorectal surgeons from 49 coloproctology units across the country using a Google Forms questionnaire. Participants were recruited through contacts from the Coloproctology Section of the Spanish Association of Surgeons and the Spanish Association of Coloproctology (AECP). The survey was sent in a personalized and blind manner, with participants unaware of others’ responses.

## Results

Sixty-two of the surveyed surgeons (71.3%) had extensive experience (over 10 years) and/or were part of AECP-accredited coloproctology units. The survey consisted of 11 questions (9 described in Table 1), with each variable (all-categorical) described in absolute numbers and percentages. Of the 101 surveyed colorectal surgeons, 87 responses were received (86.1%). Only 39 surgeons (44.8%) knew the postoperative benign stenosis rate after colon and rectal surgery. The definition of BAS was not specified in the survey, assuming the participants were familiar with it.

**Table 1:** Survey responses (*n* and %)

Do you know the rate of anastomotic stenosis after rectal and colon surgery with mechanical circular sutures?														
No	48	55.2%	Yes	39	44.8%									
According to a recent study from our center, the rate of anastomotic stenosis in the rectum after previous low resections (LAR) is 16.3% and is related to the width of the circular mechanical suture. Do you think this percentage aligns with your center/experience?														
No	46	52.9%	Yes	41	47.1%									
Do you consider this rate…?														
Too high	82	94.3%	Too low	5	5.7%									
What is the usual size of the mechanical circular suture that you use in RAB?														
28–29 mm	43	49.4%	31 mm	39	44.8%	33 mm	5	5.8%						
How do you calculate the size of the suture to be used?														
Subjectively	85	97.7%	Objectively	2	2.3%									
Do you know and/or use any mechanism that helps prevent such postoperative stenosis?														
No	63	72.4%	Yes. I know it and I use it	15	17.2%	Yes. I know it but I do not use it	9	10.4%						
Based on the current information regarding the rate of anastomotic stenosis and its relationship with the size of the circular suture, would you attempt to place a larger mechanical circular suture?														
Yes	55	63.2%	No. I don't believe in it and don't have experience	29	33.3%	No. It would waste a lot of time	3	3.5%						
What type of dilatation would you consider useful?														
Mechanical	53	Pneumatic	42	Thermal	6	Pharmacological	9	All	14					
If you had a device (laparoscopic surgery, robotic, open) that would make it easier to introduce the widest head within a maximum time of 5–10 min, would you use it?														
Yes	62	71.3%	No. I don't think it's relevant	23	26.4%	No. It’s an excessive waste of time	2	2.3%						
What price would you be willing to pay for a dilation device?														
< 10€	6	6.9%	10–50€	27	31%	50–100€	27	31%	100–200€	21	24.2%	> 200€	6	6.9%
What would be your choice of circular sutures be in case of LAR?														
25 mm	1	28–29 mm	13 11 (+ dilatator)	31 mm	22 21 (+ dilatator)	33 mm	5 14 (+ dilatator)							

In the results obtained after presenting our postoperative stenosis rate (16.3%) [10] after LAR, 41 surgeons (47.1%) considered it a similar percentage to their center or experience. Despite the balance between both options, 82 surgeons (94.3%) of those surveyed considered the rate too high.

No surgeon used 25-mm mechanical circular staplers in LAR. Forty-three (49.4%) used 28–29-mm staplers, 39 (44.8%) used 31-mm staplers, and 5 (5.7%) used 33-mm staplers. Most of them, 85 (97.7%), chose the stapler subjectively.

Sixty-three surgeons (72.4%) were unaware of the existence of mechanisms for preventing postoperative stenosis. Of the remaining percentage, only nine (10.3%) knew about them but did not use them, and 15 surgeons (17.2%) knew about and used them.

After learning the stenosis rate in our group and its relationship with the stapler diameter used, 55 professionals (63.2%) would attempt to use a larger diameter mechanical circular stapler, while 32 professionals would not—29 (33.3%) because of the lack of experience with such a high stenosis rate and 3 (3.4%) because of believing it would require too much surgical time.

If an intraoperative dilation system were available, 62 surgeons (71.3%) would use it. Of the remaining 25 (28.7%), only 2 (2.3%) would not use it, considering it an excessive waste of time during surgery, and 23 (26.4%) believed it would not be a useful device. The type of intraoperative dilation chosen varied. The most preferred method was mechanical dilation, chosen by 63 surgeons (60.9%), followed by pneumatic dilation, chosen by 42 (48.3%).

There was a disparity of opinions regarding the price surgeons would be willing to pay for an intraoperative dilation system. Sixty-four surgeons (62%) would pay between 10 and 100 euros, and only 6 (6.9%) would pay over 200 euros for the device.

Finally, in an ideal situation, half of the surgeons, 43 (49.4%), would choose 31-mm staplers; 22 (25.3%) would do so without dilation systems, and 21 (24.1%) would use dilation systems.

## Discussion

Focusing on anastomoses after rectal cancer resection, BAS is the second most serious complication. However, studies in the literature analyzing the frequency of occurrence and its associated factors are scarce. Regarding the risk factors for BAS, He et al. [11] described an association with young age, male sex, radiation therapy, protective ileostomy, intersphincteric anastomosis, distance of the anastomosis from the anal verge, and suture failure. Regarding the latter, anastomotic dehiscence is a significant risk factor for the development of postoperative stricture [4]. When there is a partial or complete dehiscence, the secondary healing process generates increased inflammation and fibrosis, which can lead to narrowing of the intestinal lumen.

For the association of BAS and the diameter of the anastomosis, to our knowledge, there is no literature regarding the impact of intraoperative dilation to achieve larger anastomotic diameters. This is likely due to a lack of awareness of their existence (as reflected in the survey presented) and their poor adaptation to modern surgery. Therefore, we consider it very important to develop an adapted device (especially for minimally invasive surgery) that allows for larger anastomotic diameters and, consequently, better surgical outcomes.

Based on the published article [8] and the current survey results, our group has developed a new device designed to facilitate the incorporation of larger diameter mechanical circular sutures, > 31 mm, in most cases, especially for laparoscopic and robotic surgery.

After a period of innovation, the device has been designed, and we are in the patenting phase (and therefore under confidentiality terms) and are in contact with the market to begin studying the utility and feasibility.

Consequently, this dilation system is a proposal that will require rigorous subsequent studies, including experimental animal models and controlled clinical trials, before it can be considered for routine clinical practice and allow for a definitive publication.

The limitations of this study are those inherent in surveys, and we acknowledge the subjective nature of the data and their wide variability depending on the centers surveyed. However, we believe that the results are of great value, as to our knowledge they represent, to date, the first confirmation of these issues in the literature.

## Conclusions

These results highlight the limited knowledge of BAS rates in LAR, its relationship with the diameter of the stapler, the subjectivity in choosing the mechanical circular stapler, and the interest in finding a solution that enables larger anastomotic diameters. Current evidence seems to suggest that, to reduce the risk of BAS, the anastomosis should be as wide as possible (31–33 mm). Although we currently do not have enough information to determine the best way to achieve the maximum anastomotic diameter, future studies with new dilation sets will attempt to address these questions and establish the optimal method for achieving a wide anastomosis.

## Data Availability

No datasets were generated or analyzed during the current study.
